# Ionic Driven Embedment of Hyaluronic Acid Coated Liposomes in Polyelectrolyte Multilayer Films for Local Therapeutic Delivery

**DOI:** 10.1038/srep14683

**Published:** 2015-10-01

**Authors:** Stephen L. Hayward, David M. Francis, Matthew J. Sis, Srivatsan Kidambi

**Affiliations:** 1 Department of Chemical and Biomolecular Engineering, University of Nebraska-Lincoln, NE, 68588; 2 Nebraska Center for Materials and Nanoscience, University of Nebraska-Lincoln, NE, 68588; 3 Mary and Dick Holland Regenerative Medicine Program, University of Nebraska Medical Center, NE, 68198.

## Abstract

The ability to control the spatial distribution and temporal release of a therapeutic remains a central challenge for biomedical research. Here, we report the development and optimization of a novel substrate mediated therapeutic delivery system comprising of hyaluronic acid covalently functionalized liposomes (HALNPs) embedded into polyelectrolyte multilayer (PEM) platform via ionic stabilization. The PEM platform was constructed from sequential deposition of Poly-L-Lysine (PLL) and Poly(Sodium styrene sulfonate) (SPS) “(PLL/SPS)_4.5_” followed by adsorption of anionic HALNPs. An adsorption affinity assay and saturation curve illustrated the preferential HALNP deposition density for precise therapeutic loading. (PLL/SPS)_2.5_ capping layer on top of the deposited HALNP monolayer further facilitated complete nanoparticle immobilization, cell adhesion, and provided nanoparticle confinement for controlled linear release profiles of the nanocarrier and encapsulated cargo. To our knowledge, this is the ***first study*** to demonstrate the successful embedment of a translatable lipid based nanocarrier into a substrate that allows for temporal and spatial release of both hydrophobic and hydrophilic drugs. Specifically, we have utilized our platform to deliver chemotherapeutic drug Doxorubicin from PEM confined HALNPs. Overall, we believe the development of our HALNP embedded PEM system is significant and will catalyze the usage of substrate mediated delivery platforms in biomedical applications.

The engineering of drug delivery platforms facilitating spatial and temporal release of a therapeutic is one of the key challenges in biomedical research that can ultimately lead to society-wide improvement in disease management. In recent years, substrate mediated delivery of cargo has shown great promise in applications including drug and gene eluding films/scaffolds^1^,^2^,^3^,^4^, coatings for stents^5^,^6^ and other implantable devices^7^,^8^, and controlling stem cell differentiation^9^. Specifically, the drug delivery kinetics is particularly relevant when it is necessary to achieve effective dose and spatiotemporal release of the therapeutic agent at the intended site of injury^10^,^11^. Delivery via immobilization of the therapeutic cargo to a solid platform demonstrates higher translatable success compared to delivery using the free “bolus” form by overcoming unfavorable burst kinetics, toxic offsite effects, and efficacy reduction due to systemic dilution. Although considerable progress has been made, there is a lack in the development of a substrate-mediated delivery system capable of simultaneous controlled and truly localized delivery of therapeutics.

In an effort to develop such sophisticated engineered surfaces, substrates such as polyelectrolyte multilayers (PEMs) have pioneered the implementation of substrate-mediated delivery in biomedical applications^12^. PEMs are an extremely robust and reproducible technique to create highly ordered polymeric thin films on a material of effectively any variety, shape, and dimension^13^,^14^,^15^,^16^,^17^,^18^,^19^,^20^,^21^,^22^,^23^,^24^. PEMs have been used to successfully deliver small molecule drugs^25^, proteins^26^, and nucleic acids^27^,^28^ by concurrently influencing cargo release kinetics and cell adhesion through specific polymer selection and overall PEM design^12^. In addition to bare therapeutics, PEMs have also been employed to encapsulate nano-drug carriers such as liposomes/micelles^29^,^30^, lipoplexes^31^, and protein based nanostructures^32^.

Liposomes have become the most widely researched, tested, and commercialized nanocarrier system. Numerous products currently on the market such as Epaxal^33^, Ambisome^34^, and Doxil^35^, with additional in clinical trials^36^, are based on a liposome delivery scheme. Due to their unique structure, liposomes are able to efficiently encapsulate and improve the pharmacokinetics of both hydrophobic and hydrophilic cargo. In addition, their high degree of freedom for surface functionalization, excellent biocompatibility, therapeutic protection during transit, and relative ease of synthesis have stimulated the development of liposome based next generation drug and gene delivery systems^37^,^38^. Several studies have demonstrated sustained release utilizing liposomes loaded with proteins or drugs and that are further incorporated into solid supports, including PEMs^39^,^40^. However, the preliminary hurdle of successfully immobilizing liposomes onto PEMs and other solid platform has drastically hindered widespread development of substrate-mediated delivery of liposome nanocarriers. Since liposomes are intrinsically fluidic due to the dynamic nature of the lipid bilayer, rapid fusion has been widely observed between liposomes and substrates during nanoparticle entrapment^41^. To overcome this, three distinct approaches have been explored: 1) surface coating of liposomes with strong polyelectrolytes (via ionic or covalent coupling) to mediate the ionic interaction with the PEM substrate and act as a steric barrier to thwart fusion^42^,^43^ 2) cholesterol modifying polyelectrolytes to interact with the hydrophobic lipid bilayer and effectively tether the nanocarrier to the substrate^44^,^45^ and 3) induced diffusion of excess polyelectrolytes to stimulate nanoparticle crosslinking to the PEM network for stabilization^46^. In all these cases, the structural integrity of the liposome is vital to the successful utilization of the platform.

Despite the salient features of PEMs and liposomes, the current research on liposome-PEM systems is extremely limited in both scope and application. Currently only few studies have analyzed the physical mechanism of liposome adsorption onto PEMs^42^,^43^, and even fewer have investigated the efficacy of such a platform in a biological setting^47^. Furthermore, no studies to date have addressed the core practicality of the liposome-PEM platform and its ability to reach a vital “therapeutic window” dependent upon loading capacity and kinetics of drug release. Herein, we report the engineering of a novel liposome (HALNP) embedded PEM substrate (HALNP-PEM) for the local and sustained delivery of therapeutic cargo. By modeling both the temporal and spatial release of our HALNP-PEM system, we have devised a novel method for sustained substrate mediated delivery of virtually any hydrophobic or hydrophilic cargo for drug delivery applications.As a model system, we utilized our platform to deliver Doxorubicin (DOX) from PEM confined HALNPs as a potent treatment option for invasive cancer. Moreover, we hypothesize a cell driven mechanism of HALNPs release from the PEM system yielding local intracellular uptake. Overall, we believe the development of our HALNP embedded PEM system is a significant advancement and will catalyze the usage of substrate mediated delivery platforms in biomedical applications and specifically the treatment for an array of disease models.

## Results

### PEM Film Fabrication and Characterization

**Figure 1:**
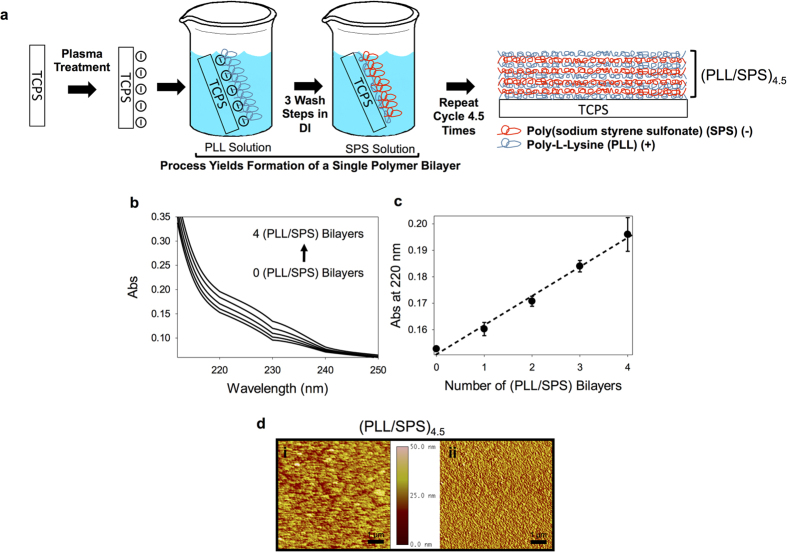
Fabrication overview, deposition validation, and characterization of the Polyelectrolyte Multilayer (PEM) base structure (PLL/SPS)_4.5_. (**a**) Fabrication Schematic for the PEM base structure utilizing layer-by-layer adsorption of PLL and SPS. Chem draw and powerpoint was used to create the drawings. (**b**) UV Spectrum Analysis of Bilayer Deposition. (**c**) UV Absorbance Readings (220 nm, characteristic for SPS) as a function of bilayer addition to validate successful base construction. (**d**) AFM of the (PLL/SPS)_4.5_ base platform via height (i) and phase contrast (ii) analysis to visually demonstrate a uniform surface ideal for nanoparticle deposition. Scale bar for AFM is 1 μm. Figure drawn by Stephen L Hayward.

To test the efficacy of a substrate mediated therapeutic delivery system implementing liposomes as an encapsulated nanocarrier, we fabricated PEM films utilizing sequential deposition of PLL and SPS (Fig. 1a) to a final design of 4.5 bilayers “(PLL/SPS)_4.5_”. We chose 4.5 bilayers because other studies have demonstrated that a similar PEM system with 4.5 bilayers produced a smooth and homogenous film^48^,^49^. PLL was selected as the polycation due to its high positive charge density, biocompatibility, cell adhesion properties, and its favorable interaction with HA that has been shown to mediate nanoparticle entrapment without rupture^43^,^50^. Analogously, SPS was selected due to its high negative charge density and vast implementation in PEM assembly^13^,^14^. The build-up of the PLL/SPS films was confirmed via UV-Vis spectrophotometry (Fig. 1b,c) using absorbance at 220 nm (finger print spectra of SPS)^51^ and fluorescence (Rhodamine tagged SPS) (Sup. Fig. 1). As illustrated in Fig. 1b,c, multilayered PLL/SPS coatings displayed linear increase in UV-absorbance spectrum with every successive double-layer addition, thus validating PEM film construction. Atomic force microscopy experimentally confirmed a high degree of surface uniformity on the (PLL/SPS)_4.5_ base platform signifying an ideal surface for nanoparticle deposition (Fig. 1d).

### Directed Deposition of HALNPs onto PEM Films

In order to incorporate liposomes into a PEM platform, a favorable interaction must occur which simultaneously immobilizes the nanoparticles and keeps their structural integrity intact. For this study, we employed a liposome system with a core bilayer structure of PC/DPPE/CHOL and an exterior coating of HA covalently bound to the surface yielding an anionic, sterically hindered nanocarrier (HALNPs). To determine the extent of the interaction between the HALNPs and PLL for potential ionic driven adsorption, we mixed the two components in PBS and measured their zeta potential and hydrodynamic diameter (Table 1). From this analysis we found even a mass ratio of 1:0.1 (Lipid: PLL) was sufficient to completely mask and reverse the charge of the HALNPs.

**Figure 2:**
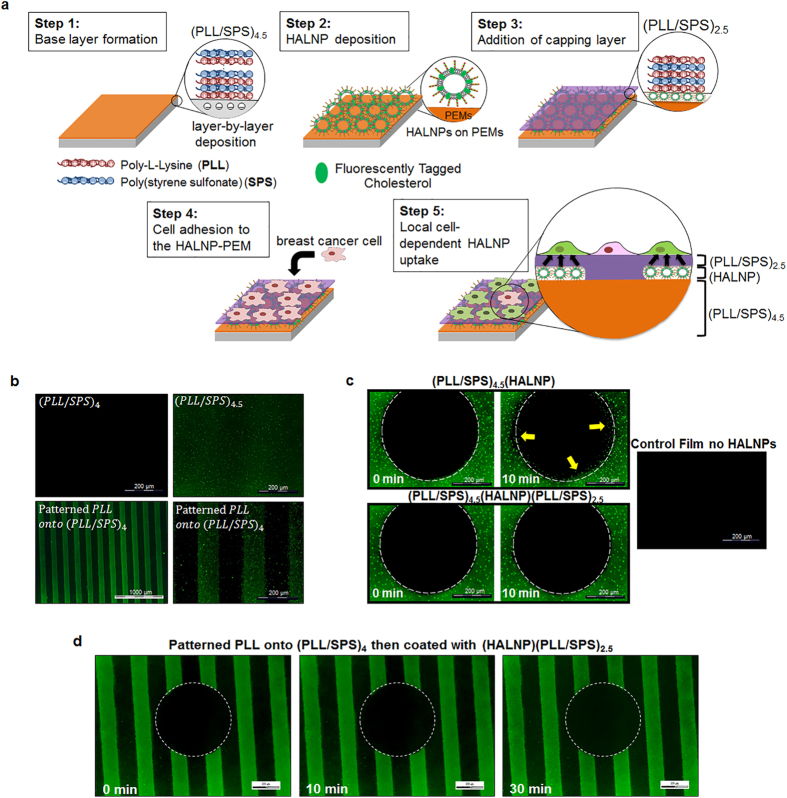
Ionic driven embedment of Hyaluronic Acid Coated Lipid Nanoparticles (HALNPs) in the PEM Platform (HALNP-PEM). (**a**) Nanoparticle embedded PEM platform overview and proposed hypothesis of action with the (PLL/SPS)_2.5_ capping layer. Chem draw and powerpoint was used to create the drawings. Figure drawn by Stephen L Hayward. (**b**) Preferential deposition of HALNPs on PLL over SPS topped surfaces. Capillary Force Lithography (CFL) was used to create PLL patterns on (PLL/SPS)_4_ and to demonstrate the level of spatial control for nanoparticle adsorption. Fluorescent Recovery after Photo bleaching (FRAP) Analysis pre and post the addition of the capping layer for 10 minutes (**c**), and with capping layer utilizing CFL for 30 minutes (**d**). The yellow arrows are pointing to particles that have moved during the experimental time lapse.

The approach for engineering the liposome embedded PEM films (HALNP-PEM) in order to develop our substrate mediated delivery platform is shown in Fig. 2a. The adsorption process of the HALNPs to polyelectrolyte surfaces was monitored by incorporation of 0.15% by mass of fluorescent cholesterol into the lipid bilayer. We first incubated the fluorescent HALNPs with both SPS (PLL/SPS)_4_ and PLL (PLL/SPS)_4.5_ topped PEM platforms and validated the selective deposition of negatively charged HALNP on positively charged PLL (Fig. 2b). To further investigate the differential adhesion of HALNP on PLL surfaces over SPS surfaces, we patterned PLL on (PLL/SPS)_4_ films using capillary force lithography (CFL) and exposed fluorescently labeled HALNPs onto these films. As observed in Fig. 2b, the HALNPs selectively adhered to the PLL regions compared to SPS films which is indicative of the fluorescent patterns formed on the PEM surfaces. We next performed a wet-phase fluorescent recovery after photo bleaching (FRAP) experiment to determine the mobility of the adsorbed particles. FRAP experiments on fluorescently tagged HALNPs deposited atop (PLL/SPS)_4.5_ films demonstrated that the HALNPs were not completely immobilized and were partially mobile on the PEM surface after 10 minutes (Fig. 2c). As a result, we increased the net immobilization of the particles via an additional ionic interaction and physical barrier in the form of a (PLL/SPS)_2.5_ “capping layer” deposited above the adsorbed HALNPs. We then re-performed the FRAP experiment on the HALNP-PEM surface with the capping layer and found that this additional barrier completely impeded the migration of the HALNPs within a PEM sandwich for both 10 min (Fig. 2c) and 30 min (Fig. 2d). Atomic force microscopy of the HALNP embedded PEM was also performed (Sup. Fig. 2). This illustrated that the adsorbed nanoparticles remained spherical pre and post addition of the capping layer, and also validated the deposition of the capping layer on top of the HALNPs.

### Loading Optimization and Release Kinetics of HALNP Immobilized in PEMs

**Figure 3:**
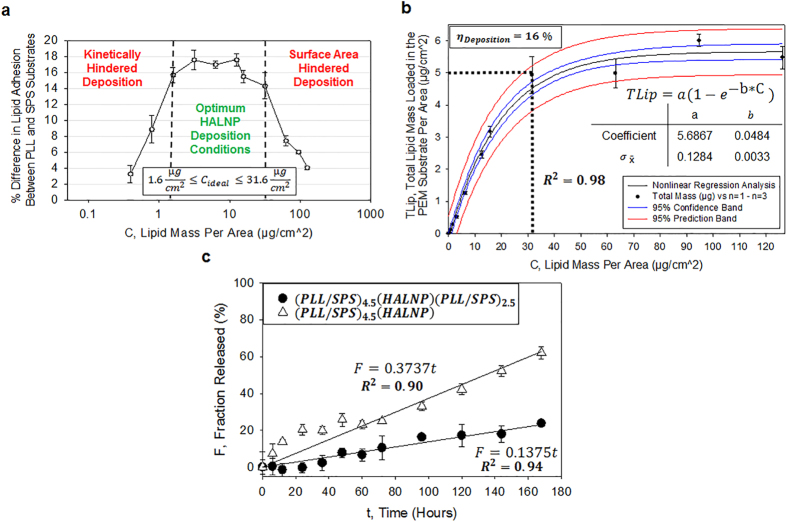
Quantitative analysis of HALNP Loading in the PEM system and subsequent release profile. (**a**) Affinity assay for HALNP absorption on (PLL/SPS)_4.5_ and (PLL/SPS)_4_ substrates to determine the optimum conditions for nanoparticle deposition. (**b**) Saturation Curve analysis of Total Lipid Loaded on (PLL/SPS)_4.5_ per square centimeter as a function of total lipid added during the three hour incubation time. (**c**) HALNP nanocarrier release profile from the non-capped [(PLL/SPS)_4.5_(HALNP)] and capped [(PLL/SPS)_4.5_(HALNP)(PLL/SPS)_2.5_] HALNP-PEM platforms.

The successful employment of the HALNP-PEM system requires the ability to deliver a known dose of drugs within a “therapeutic window” that is dependent upon release kinetics. Therefore, we first experimentally determined the optimum HALNP deposition onto the PEM base platform. We performed an affinity assay between HALNP and both (PLL/SPS)_4_ and (PLL/SPS)_4.5_ over a lipid range of 0.1 to 1000 μg/cm^2^ utilizing analogous deposition conditions described previously. By analyzing the percent difference in lipid adhesion between the PLL and SPS topped platforms over the range of lipid concentration, we estimated a minimum and maximum lipid concentration of 1.6 μg/cm^2^ and 31.6 μg/cm^2^ is necessary to overcome kinetically hindered deposition and reduction in embedment efficiency due to limited surface area, respectively (Fig. 3a). This provided us with the ideal lipid concentration range for optimum HALNP adsorption onto the PEM films. We then performed a saturation experiment analogous to a Michealis-Menton Kinetics curve to determine the maximum HALNP possible to load per unit surface area (Fig. 3b). An exponential increase followed by saturation conditions was observed for the HALNP loading with a maximum loading of 5 μg/cm^2^ HALNPs achieved. At the optimum loading concentration of 31.6 μg/cm^2^, the HALNP deposition efficiency was calculated to be 16%. This is significant information as it provides us with the ability to load a precise amount of HALNP within the PEM platform, and as a result control the cumulative total dosage of any encapsulated hydrophilic or hydrophobic cargo within the deposited lipid nanocarrier.

Next we determined the release kinetics of the HALNPs from the PEM system both with and without the capping layer (Fig. 3c). We demonstrated that approximately 60% and 20% of the adsorbed HALNPs were released in the uncapped and capped PEM systems, respectively, over the course of seven days. In addition, the capped HALNP-PEM system facilitated a linear release profile without an initial burst release that was observed in the early stages of the uncapped system. This indicated that the capping system atop the immobilized HALNPs on PEM films is the optimum platform for drug delivery as it provides a highly controlled and sustained nanocarrier release profile.

### Cellular Uptake of HALNP Immobilized in PEM Films

**Figure 4:**
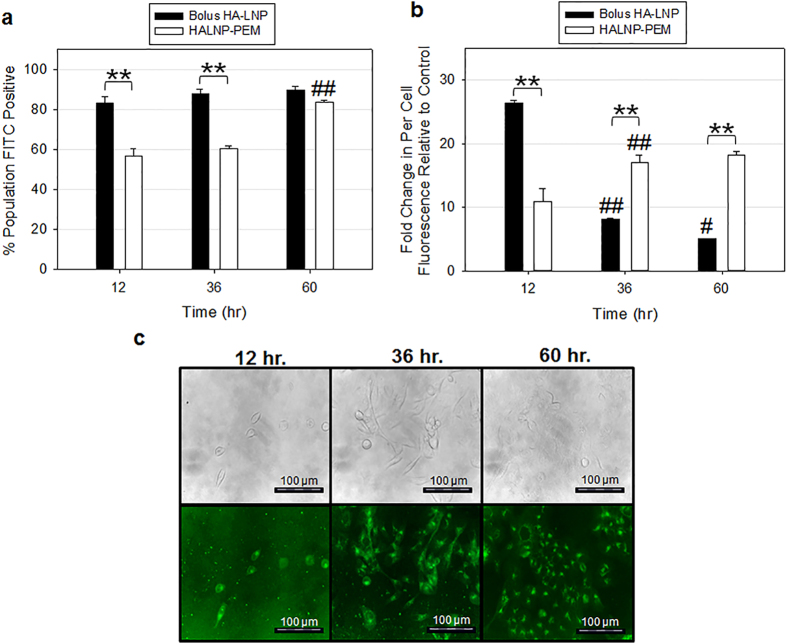
21MT-1 metastatic breast cancer cell uptake of fluorescently conjugated HALNPs from the embedded PEM system with capping layer as a function of time [(PLL/SPS)_4.5_(HALNP)(PLL/SPS)_2.5_]. Flow Cytometry Analysis of (**a**) percent cell population FITC positive and (**b**) per cell fluorescence directly comparing nanoparticle uptake between the HALNP-PEM and HALNP bolus systems (*P < 0.05, **P < 0.005; n = 3; ^#^denotes significance between a specific sample type and the preceding time point following the same significance scale as the stars). (**c**) Phase Contrast and Fluorescent Microscopy visual investigation of nanoparticle intracellular delivery on the HALNP-PEM platform.

To study the effect of nanocarrier immobilization in PEM films on cellular uptake, we compared the bolus delivery of fluorescently tagged HALNPs to the HALNPs immobilized in the PEM films with the (PLL/SPS)_2.5_ capping layer (HALNP-PEM). For this experiment, we used patient derived 21MT-1 metastatic breast cancer cells isolated from the metastatic pleural effusion as a model system. We performed a time course study via flow cytometry to determine the kinetics of cell uptake of the fluorescently tagged HALNPs when delivered in the bolus form compared to using the HALNP-PEM platform (Fig. 4a,b). At 12 hours, we found that 55% of the cells were positive for HALNP uptake from the HALNP-PEM platform compared to 81% positive cells from the bolus delivery. Also at 12 hours, the per-cell fluorescent intensity of the cells on the HALNP-PEM was 11 fold higher than control cells as compared to 26 fold higher than control for the bolus delivery. At 36 hours, the percent cell population for the HALNP-PEM system increased to 60% while the bolus delivery remained unchanged. However, the per-cell fluorescent intensity in the HALNP-PEM increased to 17 fold higher than control cells (60% increase) compared to bolus delivery that decreased to 8 fold higher than control (68% decrease). These results demonstrate that the HALNP-PEM system has a sustained release of the HALNPs while the bolus delivery system has burst release kinetics with subsequent rapid intracellular degradation. At 60 hours, 80% of the cell population was positive for HALNP uptake in the HALNP-PEM platform which is comparable to the bolus delivery at 12 hours. This confirms that the HALNP-PEM platform performs on par with the bolus delivery method in regards to the efficiency of cell uptake. Also at 60 hours the per-cell fluorescence in the HALNP-PEM system increased by 6% compared to a 40% decrease for the bolus delivery, further confirming sustained delivery with the HALNP-PEM.

To investigate the release mechanism of the HALNPs from the PEM platform, we employed fluorescent microscopy at different time intervals (Fig. 4c). We observed that the fluorescent cells increased gradually with each time interval, which is similar to the HALNP release profile demonstrated with the flow cytometry data. This experiment provided us valuable information on two important events: 1) validation of the intracellular delivery of the HALNPs and 2) evidence regarding the release of the HALNPs from the HALNP-PEM platform. The outcome of this experiment in combination with flow cytometry strongly indicated that HALNP release from the HALNP-PEM platform may be a cell-enhanced process.

### Efficacy of Embedded HALNP with Active Drug Cargo Using the HALNP-PEM System

**Figure 5:**
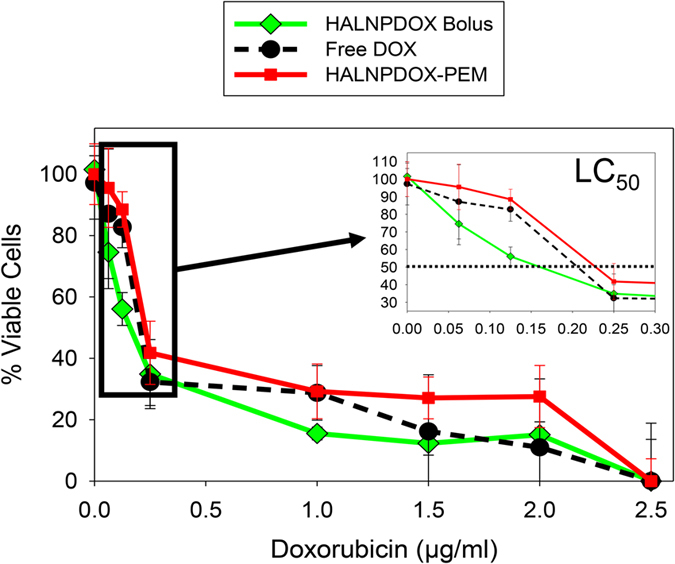
Doxorubicin potency assay comparison between Free Dox, DOX encapsulated in HALNPs (HALNPDOX) in the bolus form, and HALNPDOX nanoparticles embedded into the PEM platform (HALNPDOX-PEM). Standard MTT protocol was used to determine the % viable cells at 24 hours.

To determine the efficacy of delivering packaged drugs within the HALNP embedded nanocarrier, we immobilized HALNPs with encapsulated doxorubicin (DOX) within the HALNP-PEM platform. In addition to widespread implementation for cancer treatment, DOX was also chosen due to its extremely low molecular weight (MW) and subsequently high diffusivity with the rational that if DOX could be successfully encapsulated and embedded in a PEM platform for therapeutic purposes, virtually any hydrophilic drug could be delivered following this procedure. To compare the release mechanism and efficacy of the drug delivery, we performed a potency assay (MTT assay) on 21MT-1 metastatic breast cancer cells comparing three distinct delivery methods: 1) free DOX, 2) bolus delivery of DOX encapsulated in the aqueous core of HALNP nanoparticles (HALNPDOX Bolus), and 3) HALNPDOX embedded into the (PLL/SPS)_4.5_ base with capping layer (HALNPDOX-PEM) (Fig. 5, Table 2). To perform this comparison experiment, we first optimized the encapsulation technique of entrapping DOX inside the HALNPs, removed excess (un-encapsulated) DOX, and quantified the total internal payload of DOX via comparison to a standard curve. From this we achieved an encapsulation efficiency of 65%, with a final Lipid to DOX mass ratio of 3.125:1. We then combined this data to the amount of HALNPs that can be embedded per unit area (under saturation conditions) to precisely load a specific amount of HALNPDOX into the PEM system. Following a 24 hour incubation with 21MT-1 cells, the potency assay showed that the lowest lethal concentration to kill 50% of the cells (LC_50_) was 0.191 ± 0.030 μg/ml, 0.136 ± 0.025 μg/ml, and 0.197 ± 0.024 μg/ml for free DOX, HALNPDOX Bolus, and HALNPDOX-PEM delivery, respectively. The HALNPDOX achieved higher potency due to the potentially faster uptake by the cancer cells compared to free DOX and HALNPDOX-PEM. We also hypothesize that the HALNPDOX-PEM platform has a higher LC_50_ value compared to HALNPDOX bolus because at 24 hours not all HALNPs immobilized within the PEM films are released. As a control, a potency assay on empty HALNPs (no DOX) was performed to validate that the lipid nanocarrier is a non-toxic delivery system (Sup. Fig. 3). In summary, the DOX potency assay illustrated that the HALNP-PEM system is an ideal platform for the controlled release of active therapeutic cargo.

**Figure 6:**
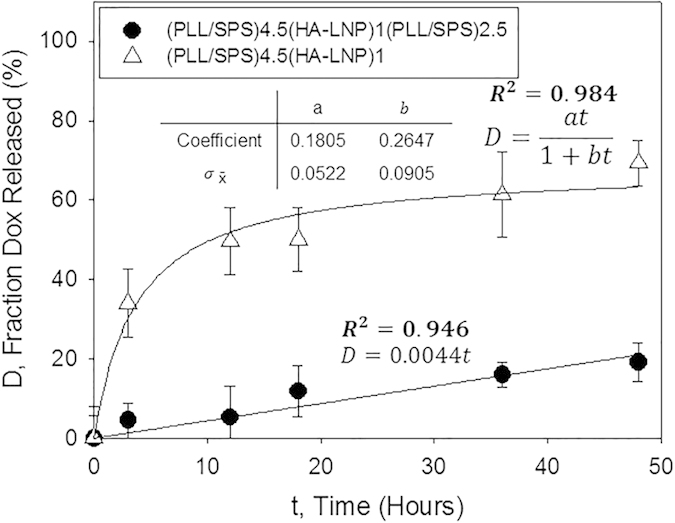
DOX release profile from the non-capped [(PLL/SPS)_4.5_(HALNP)] and capped [(PLL/SPS)_4.5_(HALNP)(PLL/SPS)_2.5_] HALNP-PEM platforms as a function of time utilizing the chemotherapeutics natural fluorescence for quantification.

We next investigated the release profile of DOX from the HALNPDOX-PEM with and without the capping layer. We monitored the release of DOX in both the supernatant and the PEM platform via drug intrinsic fluorescence as a function of time (Fig. 6). We observed that without the capping layer, DOX was released from the PEM surface in a burst release fashion leading to over 60% release by 36 hours. However, the addition of the capping layer to the HALNP-PEM platform drastically altered the DOX release kinetics and led to a linear release profile almost identical to the empty fluorescent HALNP release, signifying that the majority of DOX released was associated with a HALNP nanocarrier. This data further demonstrates that the capped PEM platform is an improved system for the controlled and linear release of therapeutic cargo.

### Spatial Control of HALNP Immobilization on the HALNP-PEM Platform Enables Local Delivery

**Figure 7:**
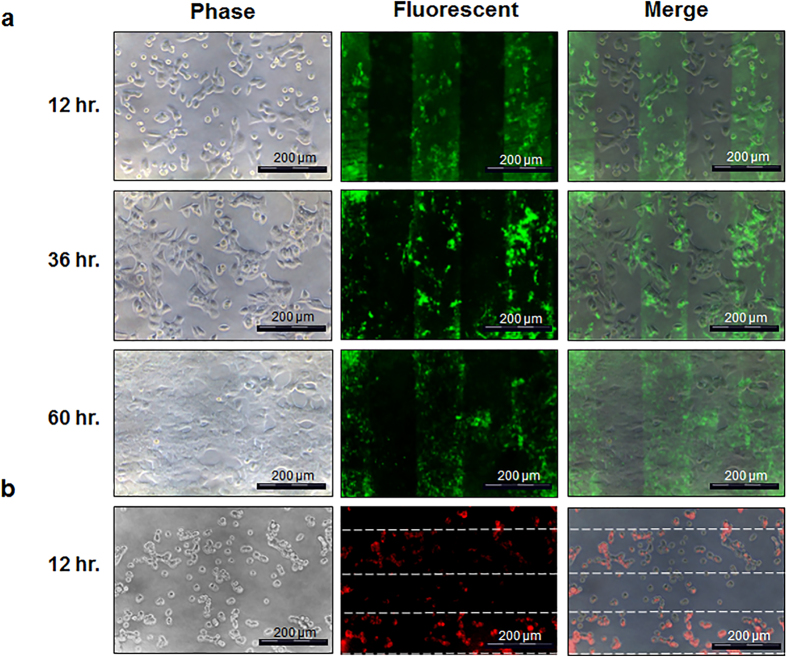
Local delivery of patterned HALNPs via the HALNP-PEM platform. Phase contrast and fluorescent microscopy images of 21MT-1 metastatic breast cancer cells adhered to the HALNP-PEM system to visually probe both the temporal and spatial release of the HALNP nanocarrier. Capillary force lithography (CFL) was used to pattern PLL and create long range order (**a**) fluorescently tagged HALNP and (**b**) HALNPDOX patterns via preferential nanoparticle adsorption.

To decipher the mechanism of HALNP release and subsequent intracellular delivery from the HALNP-PEM platform, we employed our ability to successfully pattern HALNPs to probe the spatial uptake of the nanocarrier. The principle aim of the experiment was to determine if preferential uptake will occur with 21MT-1 breast cancer cells seeded on top of the HALNP patterns compared to the cells attached on the non-HALNP areas. CFL was used to pattern HALNPs tagged with green fluorescence atop PEM base films. 21MT-1 cell adhesion to the HALNP patterned PEM system with and without the capping layer (Sup. Fig. 4) signified the necessity of the capping layer to mitigate heterogeneous cell attachment. At 12 hours post cell seeding, we observed pattern dependent cellular uptake with the cells atop the HALNP patterns having higher uptake of the particles compared to those not on the patterns (Fig. 7a). At 36 hours, the localized cellular uptake of HALNPs above the patterned layers was maintained. At 60 hours, the localization of the pattern was partially dispersed due to the proliferation and migrating metastatic breast cancer cells. This experiment further strengthened our hypothesis that the HALNP-PEM provides local delivery via a cell dependent uptake mechanism.

We also performed this experiment with DOX encapsulated inside the patterned HALNP-PEM system to determine if the local delivery of an active therapeutic was also possible with our liposome embedded PEM platform. We first adsorbed HALNPDOX onto the patterned PLL followed by subsequent addition of the capping layer. 12 hours after seeding of 21MT-1 cells, we observed a local uptake of the HALNPDOX particles dependent upon the nanoparticle deposition pattern (Fig. 7b). We selected a time point of 12 hours to demonstrate the spatial release and uptake of DOX by the cells residing atop the HALNP embedded regions to ensure the cells were still alive to illustrate this phenomena. When comparing the HALNPDOX uptake profile to the empty nanocarrier itself, the degree of control over the cell uptake is slightly hindered due to the high diffusion and extensive cell uptake of free DOX. Nonetheless, by using DOX and demonstrating this level of spatial and temporal control can be achieved validates the application of the HALNP-PEM platform for use in an extremely broad range of localized therapeutic delivery.

## Discussion

Surface-mediated drug delivery involves the administering of therapeutics to adhering or suspension cells from either implantable devices or from tissue engineering scaffolds. Recently, liposomes have gained attention as a promising tool for targeted drug delivery to various parts of the body including tumors^37^,^38^,^39^,^40^. Several preclinical studies using drug-encapsulated liposomes have shown improvement in the sustained release of the drug, prolongation of the drug’s half-life, and an increase in the therapeutic index for drugs delivered *via* this route^37^,^38^,^39^,^40^. The surface immobilization of liposomes still remains a challenge and has drastically hindered development of substrate-mediated delivery of liposome nanocarriers. Thus far, liposome containing PEMs have been demonstrated to successfully 1) deliver fluorescent lipids to adhered cells in a polymer composition and bilayer dependent manner^44^,^52^, 2) coat microneedles for transdermal vaccine administration^53^, and 3) deliver small hydrophobic cytotoxic compound to adhered cells^44^. However, these studies have not addressed the core practicality of the liposome-PEM platform and its ability to reach a vital “therapeutic window” dependent upon loading capacity and kinetics of drug release.

Here, we report the development of a novel substrate mediated therapeutic delivery system comprising of liposomal nanoparticles (LNPs) covalently functionalized with hyaluronic acid (HALNPs) and embedded within a PEM platform (HALNP-PEM) via ionic interactions. Both affinity assay and saturation kinetic curves were performed to determine the optimum HALNP embedment conditions and the maximum HALNP embedment capacity per unit area. A capping layer of 2.5 additional bilayers of (PLL/SPS) on top of the HALNP monolayer was employed for complete nanoparticle immobilization, support of cell adhesion, and increased control over the release profile of both the HALNP carrier and any therapeutic encapsulated in the nanocarrier. We directly compared bolus HALNP and the HALNP embedded within the PEM films to quantify the result of liposome confinement on intracellular uptake kinetics in patient derived metastatic breast cancer cells (21MT-1) isolated from the metastatic pleural effusion. This experiment validated the sustained release profile of over 60 hours for the HALNP-PEM system as compared to the burst release kinetics of the bolus HALNPs. In addition, we chose Doxorubicin (DOX) as a model hydrophilic cargo to verify the structural integrity of the particles following deposition and to probe the therapeutic index of the HALNP-PEM platform.

We also analyzed the mechanism of HALNP uptake from the PEM platform. While controlled release of the HALNP was observed in both a cell containing (Fig. 4a,b), and a cell-free system (Fig. 3c, **capped**), the time scale of release was significantly different in these two scenarios. For the cell-free experiment, at the 60 hour time point less than 10% of the total HALNP loaded into the PEM was released by diffusion/film swelling alone. However, for the cell containing experiment the flow cytometry data indicates that at the 60 hour time point all particles have been released in order to achieve the percent population positive for HALNP uptake seen in the bolus delivery at the 12 hour time point (100% uptake). In order for both of these events to hold true, there must be a strong driving force for HALNP release mediated by the cells themselves. To decipher this HALNP release mechanism from the PEM, we employed capillary force lithography (CFL) to pattern either fluorescently conjugated HALNPs or DOX encapsulated within HALNPs into our PEM platform. This time course study revealed the local cell-dependent uptake of HALNPs from the HALNP-PEM system - only cells above the patterned region took up the fluorescent nanoparticles. These findings further strengthened our hypothesis that release is driven by a cell-dependent uptake mechanism.

This study has systematically demonstrated the successful embedment of a proven translatable lipid based nanocarrier, HALNPs, into a PEM system via ionic interaction. This HALNP-PEM platform was capable of local and sustained delivery of a hydrophobic cargo in the form of fluorescently tagged cholesterol and a hydrophilic cargo in the form of the widely utilized chemotherapeutic, Doxorubicin. We modeled the maximum loading capacity of HALNPs per unit area for precise loading of any therapeutic cargo type, probed the HALNP uptake mechanism from the PEM system via implementation of the soft lithography technique CFL and found a cell mediated uptake process, and validated the ability of the HALNP system to retain the efficacy of an active therapeutic, DOX, all while controlling the spatiotemporal release profile.

Overall, the development of our HALNP embedded PEM system will catalyze the usage of substrate mediated delivery platforms in biomedical applications, including the treatment of an array of disease models. For example, the current treatment method for Glioblastoma Multiforme (GBM) is maximal surgical resection of the cancerous tissue, radiotherapy, and chemotherapy^54^. We believe our substrate delivery platform has the potential to develop an implantable device that can occupy the cavity of the resected tumor and promote local and sustained delivery of HALNPs containing either chemotherapeutics^55^ or silencing RNAs^56^ for long term GBM mangement. Furthermore, although this study utilized cancer and the popular chemotherapeutic DOX as a model system, the HALNP-PEM platform could also be employed in a variety of other areas such as wound healing to locally delivery growth factors, anti-microbial, or supplements to aid in healing advancement^57^, liposomal mediated drug and/or gene delivery from stents^5^,^6^,^28^, and next generation transcutaneous vaccine delivery^53^,^58^. The development of the HALNP-PEM system is a significant improvement over current substrate mediated drug delivery platforms and will thus have a far reaching impact in biomedical research as a whole.

## Materials and Methods

***Please refer to the supplemental information for the full Materials and Methods.

### PEM Base and HALNP Fabrication

A polyelectrolyte multilayer (PEM) base structure of PLL and SPS was constructed by sequential adsorption of the polyelectrolytes on top of an oxygen plasma treated surface (glass slide or tissue culture plate). Multilamellar vesicles (MLVs) composed of PC, DPPE, and CHOL in a (3:1:1) molar ratio were created by the traditional dry film method as previously reported^59^,^60^. The MLV solution was then extruded through progressively smaller membrane pore sizes, purified by ultracentrifugation (1.5 hr., 135,000 g) to remove lipid debris, and covalently cross-linked to high molecular weight hyaluronic acid (HA) via EDC mediated amide bond formation during an overnight incubation at 37 °C. Separation of the resulting HALNPs from excess reagents in solution was achieved by washing three times using ultracentrifugation. Following purification, the particles were aliquoted, snap frozen in ethanol with dry ice, and lyophilized using a Labconco Chamber Freeze Dry System (Kansas City, MO, USA). The lyophilized particles were stored at −80 °C until use.

### Adsorption Process for HALNPs

In order to track the adsorption of HALNPs, 0.15 mass % of Top Fluor fluorescently conjugated cholesterol was added to the initial lipid stock to effectively tag the nanoparticles. Then, to initially determine if the HA-LNPs could successfully adsorb onto a PEM surface, 180 μg lipid/cm^2 was incubated with both PLL (PLL/SPS)_4.5_ and SPS (PLL/SPS)_4.5_ topped base layers fabricated in a 12 well plate for three hours at room temperature. Following incubation, the surfaces were washed 3× with 20 mM HEPES and visualized with an Axiovert 40 CFL Zeiss (Jena, Germany) Inverted Microscope with fluorescent filter, and the images were captured with a Progres C3 Jenoptick camera. Following deposition of the HALNPs, a capping layer consisting of an addition 2.5 bilayers of PLL and SPS, (PLL/SPS)_2.5_, was added on top of the anionic nanoparticle monolayer following previously described adsorption protocol with the sole procedure change of using 20 mM HEPES for wash steps in place of DI water. The capping layer was added sequentially by hand in an aseptic environment to ensure sterile conditions.

### Affinity Assay and Saturation Curve for HALNP Loading

To evaluate the loading of HALNPs into the PEM platform a dual affinity and saturation curve assay was systematically implemented to determine optimum and saturated deposition conditions. First, a standard curve of known fluorescent HALNPs mass (termed lipid mass) was used to create a fluorescent standard curve. Then, two 48 well plates were coated with a PEM base layer. One plate was coated with a PLL topped layer (PLL/SPS)_4.5_, while the other was coated with an SPS topped layer (PLL/SPS)_4_. Then, a range HALNP concentration, 1 to 800 μg/ml, was incubated with the 48 well plates for 3 hours at room temperature covered in foil. Following this incubation time, the supernatants from each well were read against the standard curve to determine concentration of un-attached nanoparticles, and the percent difference between the PLL and SPS topped substrate wells (at analogous nanoparticle incubation concentration) was used to determine the affinity of the HALNPs for that particular surface. The concentration of embedded particles deposited on the plate was also measured by reading the 48 well plate in a Biotek Synergy 2 Multi-Mode Plate Reader (Winooski, VT, USA) to confirm conservation of mass (ex. 495 nm, em. 520 nm). To determine the HALNP saturation conditions of the PLL topped base platform, the 1) supernatant and 2) nanoparticle embedded plate data from the affinity assay was used to determine the exact amount of mass loaded per area as a function of the nanoparticle loading concentration. From this information, the saturation point of HALNPs was determined from a nonlinear regression analysis via Sigma Plot Software (San Jose, CA, USA).

### Flow Cytometry

Flow cytometry was performed using a FACSCantoII from Becton Dickenson (Franklin Lakes, NJ, and USA). Flow cytometry was specifically employed because it gives both per cell and population wide assessment of nanoparticle uptake via gating the analyzed cells into either a FITC positive (positive for HALNP uptake) or a FITC negative (no HALNP uptake) domain. A 12 well culture plate was first plasma treated and deposited with a PEM base of (PLL/SPS)_4.5_. This base platform was then UV sterilized overnight. Filter sterilized HALNPs with 0.15% Top Fluor fluorescent cholesterol were then deposited on the PLL surface applying saturation conditions, followed by the addition of the capping layer (5 μg/cm^2^; total HALNP loading in a 12 well = 20.05 μg HALNP per well). Each well was then seeded with 170,000 21MT-1 cells and placed in the incubator overnight to promote cell attachment. At 12, 24, and 36 hrs. The cells were washed 3× with sterile 1× PBS, trypsinized, and transferred to flow cytometry tubes. Theses samples were then analyzed for fluorescence in the green channel (ex. 495 nm, em. 520 nm; 10,000 total events/read) against control cells. Both per cell fluorescence and total % population FITC positive information was measured. Additionally, bolus form HALNPs, free nanoparticles not bound to a substrate, were also incubated with 21MT-1 cells in an analogous manner (same seeding density of cells and concentration of 20.05 μg HALNPs per well) to compare the substrate mediated and bolus form intracellular delivery of HALNPs as a function of time.

### Potency Assay

The DOX concentration lethal to 50% of the 21MT-1 cells (LC_50_) was determined utilizing the MTT (3-(4,5-dimethyldiazol-2-yl)2,5 diphenyl Tetrazolium Bromide) assay kit from Life Technologies (Carlsbad, CA, USA). This classical colorimetric assay assesses cell health as a function of the mitochondrial conversion of MTT salt to Formazan. A two-step procedure was implemented for precise loading of DOX into the HALNPs and then into the HALNP-PEM platform. First a solution of 500 μg of DOX in 0.05× PBS was used to rehydrate a lyophilized vial of HALNPs and excess (un-encapsulated) DOX was removed using ultracentrifugation (140,000 g, 1.25 hrs, 4 Deg C). A standard curve of known DOX concentrations was employed to determine the total amount of DOX encapsulated in the HALNP sample (“HALNPDOX”). The initial vial of HALNPs (1 mg Total Lipid) concentration was known, thus both the total amount of encapsulated DOX and lipid were fully determined. Secondly, the HALNPDOX particles were adsorbed onto the (PLL/SPS)_4.5_ base platform ensuring the amount of lipid required to achieve a specified dosage of DOX was below the saturation conditions of the lipid as seen in Fig. 3b. The potency assay was run between 2.5 μg/ml DOX to 0 μg/ml DOX. Following purification, the final mass ratio between Lipid and DOX was 3.125:1. Therefore, for the 2.5 μg/ml DOX concentration samples (highest concentration tested in potency assay), 3.9 μg HALNPDOX was adsorbed per well to achieve the desired DOX concentration (well volume = 0.5 ml). This similar reasoning was used for all lower concentrations of HALNPDOX loading as well. The only difference in this loading procedure compared to empty HALNPs was the increase in the deposition incubation time from three hours at room temperature to overnight at 4 °C to ensure complete embedment of the HALNPDOXs as well as protect the activity of the DOX. Supernatant samples were removed following the adsorption protocol to ensure all particles deposited onto the (PLL/SPS)_4.5_ base platform. In addition, DLS and Zeta potential analysis of the HALNPDOX particles was analyzed to ensure the encapsulation of the DOX did not significantly alter with the size or charge of the HALNPs (and therefore the adsorption process). The (PLL/SPS)_2.5_ capping layer was added immediately after the HALNPDOX adsorption.

21MT-1 cells were seeded at a density of 32,000 cells/well in three 48 well plates with different DOX configurations: 1) PEM platform with embedded HALNPDOX [(PLL/SPS)_4.5_(HALNPDOX)(PLL/SPS)_2.5_], 2) Bolus form HALNPDOX, and 3) free non-encapsulated DOX. After a 24 hours incubation time between the three systems and the 21MT-1 cells, the media was aspirated and 5 mg/ml MTT working solution was added and incubated for 2 hours at 37 °C. Cells were then lysed with lysis buffer (acidified IPA) and the absorbance was measured at 570 and 620 nm using a Beckman Coulter AD340 plate reader (Indianapolis, IN, USA). Percent viability was determined by normalization of the 570/620 absorbance ratio to the control untreated cells and positive control dead cells.

***Please refer to the supplemental information for detailed methods.

## Additional Information

**How to cite this article**: Hayward, S. L. *et al.* Ionic Driven Embedment of Hyaluronic Acid Coated Liposomes in Polyelectrolyte Multilayer Films for Local Therapeutic Delivery. *Sci. Rep.* **5**, 14683; doi: 10.1038/srep14683 (2015).

## Supplementary Material

Supplementary Information

## Figures and Tables

**Table 1:** Dynamic Light Scattering and Zeta Potential Analysis of the LNP systems.

Sample ID (Lipid:PLL mass ratio)	Hydrodynamic Diameter (nm)	Polydispersity Index	Zeta Potential (mV)
LNP	95.1 ± 0.6	0.087	−9.46 ± 0.31
HALNP	164.9 ± 1.5	0.198	−23.29 ± 4.70
HALNPDOX	175.9 ± 7.5	0.233	−17.05 ± 2.40
HALNP-PLL (1:0.1)	235.2 ± 8.5	0.278	20.08 ± 0.42
HALNP-PLL (1:0.5)	310.6 ± 19.0	0.310	22.74 ± 0.64
HALNP-PLL (1:1.0)	363.0 ± 31.0	0.360	23.45 ± 0.62

**Table 2:** Potency assay summary (LC_50_ values) between the free form DOX, bolus HALNPDOX, and HALNPDOX-PEM substrate to 21MT-1 Metastatic Breast Cancer Cells at 24 hrs.

Delivery Method	LC_50_ (μg/mL)
Free DOX	0.191 ± 0.0301
HALNPDOX Bolus	0.136 ± 0.0248
HALNPDOX-PEM	0.197 ± 0.0237
