# Supplementary material for: Ionic Driven Embedment of Hyaluronic Acid Coated Liposomes in Polyelectrolyte Multilayer Films for Local Therapeutic Delivery
Source: Sci Rep. 2015 Oct 1;5:14683. doi: 10.1038/srep14683 (PMC4589783; doi:10.1038/srep14683)
Supplement: Supplementary Information [file srep14683-s1.docx]

**Ionic Driven Embedment of Hyaluronic Acid Coated Liposomes in Polyelectrolyte Multilayer Films for Local Therapeutic Delivery**

Stephen L. Hayward^1^, David M. Francis^1^, Matthew J. Sis^1^, & Srivatsan Kidambi^1, 2, 3#^

^1^Department of Chemical and Biomolecular Engineering,

University of Nebraska-Lincoln, NE, 68588

^2^Nebraska Center for Materials and Nanoscience,

University of Nebraska-Lincoln, NE, 68588

^3^Regenerative Medicine Program,

University of Nebraska Medical Center, NE, 68198.

^#^indicates corresponding author. Email: [skidambi2@unl.edu](mailto:skidambi2@unl.edu)

**Supplementary Information**

**Expanded Materials and Methods**

**Materials.** For Polyelectrolyte Multilayer (PEM) fabrication, Poly-L-Lysine (PLL) hydrochloride with a molecular weight greater than 30,000 Da and Poly(sodium styrene sulfonate) (SPS) with a molecular weight of ~ 70,000 Da were purchased from Sigma Aldrich (St. Louis, MO, USA). For liposome assembly and nanoparticle surface conjugation, Hyaluronic Acid (HA) with a molecular weight of 1.65 MDa, 1, 2- Dipalmitoyl-sn-Glycero-3-Phopshoethanolamine (DPPE), Cholesterol (CHOL), and 1-ethyl-3-(3-dimethylaminopropyl) carbomiide (EDAC) were purchased from Sigma. Additionally, L α-Phosphatidylcholine (PC) and Top Fluor fluorescently tagged cholesterol were purchased from Avanti Polar Lipids (Alabaster, AL, USA). For Capillary Force Lithography (CFL), PDMS elastomer (SYLGARD 184 Silicone Elastomer Base) and PDMS cross linker (SYLGARD 184 Silicone Elastomer Curing Agent) were purchased from Dow Corning Corporation (Midland, MI, USA). Fluorosilane (Trichloro (3, 3, 3-trifluoropropyl) silane) was purchased from Sigma.

**Polyelectrolyte Multilayer Base Fabrication.** A polyelectrolyte multilayer (PEM) base structure of PLL and SPS was constructed by sequential adsorption of the polyelectrolytes on top of an oxygen plasma treated surface (glass slide or tissue culture plate). Briefly, the negatively charged surface was first incubated with 0.5 mg/ml PLL (20 μM PBS, 0.15M NaCl) for 20 minutes, followed by numerous DI washing steps to remove excess polymer, and subsequent incubation with 10mM SPS (0.1M NaCl) for another 20 min to create a single PEM bilayer. This process was performed 4.5 times to create the PLL topped PEM base structure (PLL/SPS)_4.5_$.$When used for cell culture experiments, the PEM base structure was UV sterilized for 8-12 hours in a biosafety cabinet.

**Preparation of HA coated Liposomes (HALNPs).** Multilamellar vesicles (MLVs) composed of PC, DPPE, and CHOL in a (3:1:1) molar ratio were created by the traditional dry film method as previously reported ^1-6^. The MLV solution was then extruded through progressively smaller membrane pore sizes with a mini extruder apparatus (Avanti Polar Lipids) kept above the gel transition temperature of the lipids to reach a final hydrodynamic diameter of ~100 nm (LNP). These nanoscale LNPs were purified by ultracentrifugation (1.5 hr., 135,000 g) to remove lipid debris and were re-suspended in borate buffer (pH 8.6). To covalently coat the nanoscale LNPs, HA was dissolved in sodium acetate buffer (pH 5), activated with EDAC at a mass ratio of 1:20 (HA:EDAC) at 37°C (pH ~4) for 2 hours ^7^, and mixed with the purified LNPs overnight to promote amide bond formation (HALNPs). Separation of the resulting HALNPs from excess reagents in solution was achieved by washing three times using ultracentrifugation. Following purification, the particles were aliquoted, snap frozen in ethanol with dry ice, and lyophilized using a Labconco Chamber Freeze Dry System (Kansas City, MO, USA)^6^. The lyophilized particles were stored at -80 °C until use.

**Dynamic Light Scattering and Zeta Potential Analysis of the Nanocarrier System.** Mean hydrodynamic diameter and zeta potential were measured using a Brookhaven NanoBrook ZetaPALS zeta potential and dynamic light scattering instrument (Holtsville, NY, USA). The particle size was analyzed as an intensity averaged distribution using a scattering angle of 90° at 25°C. The Smoluchowki model was utilized to calculate the zeta potential from mobility measurements. All measurements were performed in 0.05x PBS (pH 7.4) at 25°C.

**Adsorption Process for HALNPs.** In order to track the adsorption of HALNPs, 0.15 mass % of Top Fluor fluorescently conjugated cholesterol was added to the initial lipid stock to effectively tag the nanoparticles. Then, to initially determine if the HA-LNPs could successfully adsorb onto a PEM surface, 180 μg lipid/cm^2 was incubated with both PLL (PLL/SPS)_4.5_ and SPS (PLL/SPS)_4.5_ topped base layers fabricated in a 12 well plate for three hours at room temperature. Following incubation, the surfaces were washed 3x with 20 mM HEPES and visualized with an Axiovert 40 CFL Zeiss (Jena, Germany) Inverted Microscope with fluorescent filter, and the images were captured with a Progres C3 Jenoptick camera. Following deposition of the HALNPs, a capping layer consisting of an addition 2.5 bilayers of PLL and SPS, (PLL/SPS)_2.5_, was added on top of the anionic nanoparticle monolayer following previously described adsorption protocol with the sole procedure change of using 20mM HEPES for wash steps in place of DI water. The capping layer was added sequentially by hand in an aseptic environment to ensure sterile conditions.

**Affinity Assay and Saturation Curve for HALNP Loading.** To evaluate the loading of HALNPs into the PEM platform a dual affinity and saturation curve assay was systematically implemented to determine optimum and saturated deposition conditions. First, a standard curve of known fluorescent HALNPs mass (termed lipid mass) was used to create a fluorescent standard curve. Then, two 48 well plates were coated with a PEM base layer. One plate was coated with a PLL topped layer (PLL/SPS)_4.5_, while the other was coated with an SPS topped layer (PLL/SPS)_4_. Then, a range HALNP concentration, 1 to 800 μg/ml, was incubated with the 48 well plates for 3 hours at room temperature covered in foil. Following this incubation time, the supernatants from each well were read against the standard curve to determine concentration of un-attached nanoparticles, and the percent difference between the PLL and SPS topped substrate wells (at analogous nanoparticle incubation concentration) was used to determine the affinity of the HALNPs for that particular surface. The concentration of embedded particles deposited on the plate was also measured by reading the 48 well plate in a Biotek Synergy 2 Multi-Mode Plate Reader (Winooski, VT, USA) to confirm conservation of mass (ex. 495 nm, em. 520 nm). To determine the HALNP saturation conditions of the PLL topped base platform, the 1) supernatant and 2) nanoparticle embedded plate data from the affinity assay was used to determine the exact amount of mass loaded per area as a function of the nanoparticle loading concentration. From this information, the saturation point of HALNPs was determined from a nonlinear regression analysis via Sigma Plot Software (San Jose, CA, USA).

**Fluorescent Recovery after Photobleaching (FRAP).** A HALNP loading of 5 μg lipid/cm^2 was deposited onto a (PLL/SPS)_4.5_ base layer utilizing the previously mentioned saturation protocol. Then, a portion of the deposited HALNP layer was photo bleached with an Axiovert 40 CFL Zeiss (Jena, Germany) Inverted Microscope with a FITC filter for 5 minutes.^8^ The fluorescent microscope was then shut off for a designated amount of time (10 or 30 minutes) to allow for the assessment of nanoparticle mobility. After this wait time, the microscope was quickly turned back on and a fluorescent image was immediately captured. This procedure was performed both with and without a (PLL/SPS)_2.5_ capping layer over the HALNP deposited monolayer to compare the effect of nanoparticle confinement on mobility. All analyses were performed in wet conditions, in 20 mM HEPES buffer at pH 7.4 at room temperature. A control film without labeled nanoparticles has also been included to discern any background fluorescence.

**Atomic Force Microscopy (AFM).** AFM was used to visualize the PEM base platform pre and post deposition with HALNPs. To do so, three samples were created: 1) the PEM base platform (PLL/SPS)_4.5_ , 2) HALNPs deposited on the base platform without capping layer, and 3) deposited HALNPs on the (PLL/SPS)_4.5_ base with a a (PLL/SPS)_2.5_ capping layer. All samples were allowed to air dry in an oven at 25°C overnight. The AFM analysis was performed with a Nanocscope IIIa Dimension 3100 SPM system (Digital Instruments, Santa Barbara, CA) operating in tapping mode. This instrument is located in the Nebraska Center for Materials and Nanoscience’s Scanning Probe Microscopy. Both height and phase (deflection) images were obtained.

**21MT-1 Cell Culture.** 21MT-1 Cells were a kind gift from Dr. Band at the University of Nebraska Medical Center. This cell line was isolated from the metastatic pleural effusion mammary tumor specimens. The 21MT-1 cells were cultured in α-MEM media supplemented with 1% Penicillin-Streptomycin (PS), 1% L-glutamine, 20 mM HEPES, 5% fetal bovine serum (FBS), non-essential amino acids, sodium pyruvate (all listed reagents from Invitrogen), 12.5 ng/ml epidermal growth factor (EGF) and 1 µg/ml hydrocortisone (both from Sigma). The cells were kept in aseptic conditions, and grown in an incubator at 37°C with 5% CO2.

**Capillary Force Lithography (CFL) to pattern HALNPs.** The silicone master pattern was secured to the bottom of a large cell culture dish, and was placed in a desiccator with an open vial of fluorosilane. The desiccator was evacuated by vacuum pump, creating a partial pressure of fluorosilane. Meanwhile, PDMS elastomer and cross linker were combined in a (9:1) mass ratio, and stirred vigorously by hand for five minutes. The silicone master pattern was removed from the desiccator, and the PDMS mixture was poured over the master pattern. The PDMS covered master pattern was placed in a new desiccator, which was alternately evacuated by vacuum pump and re-pressurized to remove any bubbles in the liquid PDMS. Once all bubbles were eliminated, the mixture was cured in the oven at 60°C for 24 hours. After curing, the PDMS was removed from the silicone master pattern with a scalpel, and the stamps were cut to size.

PDMS micro-patterned line stamps were cut into rectangular sections with open channels on two out of four faces. The stamps were cleaned with liquid hand soap and DI water, and then blown dry with air. They were then treated in an oxygen plasma cleaner for one minute in order to induce a negative charge on their surface. The stamps were then placed pattern down on top of polyelectrolyte multilayer (PEM) coated glass slides (PLL/SPS)_4_, and stabilized with a weight (90g) placed on top. Ten μL of 0.5 mg/mL PLL solution was evenly applied to the edge of the stamp with a pipette, at the contact point of the open channels and the PEM coated slide. By capillary force and charge/charge interactions, PLL solution was drawn into the channels. This specific method of capillary force lithography is commonly referred to as Micromolding in Capillaries.^9^ The patterns were allowed to dry for twenty-five minutes before the stamps were removed. Following this dry time the patterned PLL was UV sterilized for 8-12 hours, coated with HALNPs, and capped with the capping layer prior to cell culture work.

**Flow Cytometry.** Flow cytometry was performed using a FACSCantoII from Becton Dickenson (Franklin Lakes, NJ, and USA). A 12 well culture plate was first plasma treated and deposited with a PEM base of (PLL/SPS)_4.5_.This base platform was then UV sterilized overnight. Filter sterilized HALNPs with 0.15% Top Fluor fluorescent cholesterol were then deposited on the PLL surface applying saturation conditions, followed by the addition of the capping layer (5 µg/cm^2^; total HALNP loading in a 12 well = 20.05 µg HALNP per well). Each well was then seeded with 170,000 21MT-1 cells and placed in the incubator overnight to promote cell attachment. At 12, 24, and 36 hrs. The cells were washed 3x with sterile 1x PBS, trypsinized, and transferred to flow cytometry tubes. Theses samples were then analyzed for fluorescence in the green channel (ex. 495 nm, em. 520 nm; 10,000 total events/read) against control cells. Both per cell fluorescence and total % population FITC positive information was measured. Additionally, bolus form HALNPs, free nanoparticles not bound to a substrate, were also incubated with 21MT-1 cells in an analogous manner (same seeding density of cells and concentration of 20.05 µg HALNPs per well) to compare the substrate mediated and bolus form intracellular delivery of HALNPs as a function of time.

**Encapsulation of Doxorubicin (DOX) in HALNPs (HALNPDOX).** Vials of lyophilized HALNPs were brought to room temperature and rehydrated with 1/10^th^ of the original pre-lyophilized volume with DOX dissolved in 0.05x PBS. These vials were then quickly vortexed to hydrate the entire dry lipid film, and left to rest for 30 minutes on a shaker table to mediate lipid membrane re-assembly and optimum DOX entrapment (HALNPDOX). Following the entrapment procedure, additional 1x PBS was added to bring the vials to the pre-lyophilized volume. Un-encapsulated DOX was removed by ultracentrifugation (140,000 g, 4°C, and 1.25 hr.) and subsequent wash steps with 1x PBS. To determine the amount of encapsulated chemotherapeutic in the aqueous core of the HALNPs, the natural fluorescence of DOX (ex. 470 nm, em. 585 nm) was measured in the presence of 0.1 % Triton X-100 detergent to disrupt the lipid bilayers and compared to a standard curve of known DOX concentration. Following excess DOX removal, and quantification of total internal DOX payload, the final mass ratio between Lipid (HALNP) and DOX was 3.125:1. Therefore, the max loading of DOX encapsulated into the aqueous core of the HALNPs and then subsequently loaded onto the (PLL/SPS)_4.5_ based on lipid saturation of the PEM is 1.6 µg DOX/cm^2^.

**Cargo (DOX) and Carrier (HALNP) Release Kinetics from the PEM platform.** The kinetics of release for the HALNP nanocarrier from the PEM platform with and without a (PLL/SPS)_2.5_ capping layer was assessed by employment of a fluorescent plate reader. The PEM system embedded with HALNPs was incubated in 1xPBS at room temperature. At specific time points, the nanoparticle release medium was replaced to acquire samples and to ensure infinite sink conditions were maintained. Both supernatant and the HALNP embedded plate data (utilizing the established saturation conditions) were analyzed to confirm conservation of mass (ex. 495 nm, em. 520 nm). Release kinetics of HALNP encapsulated DOX from the capped and non-capped PEM platform was performed in the analogous manner utilizing the natural fluorescence of the chemotherapeutic (ex. 470 nm, em. 585 nm) in comparison to a standard curve.

**Potency Assay.** The DOX concentration lethal to 50% of the 21MT-1 cells (LC_50_) was determined utilizing the MTT (3-(4,5-dimethyldiazol-2-yl)2,5 diphenyl Tetrazolium Bromide) assay kit from Life Technologies (Carlsbad, CA, USA). This classical colorimetric assay assesses cell health as a function of the mitochondrial conversion of MTT salt to Formazan. A two-step procedure was implemented for precise loading of DOX into first the HALNPs and then into the PEM platform. First a solution of DOX was used to rehydrate a lyophilized vial of HALNPs, excess (un-encapsulated) DOX was removed, and a standard curve of known DOX concentrations was employed to determine the total amount of DOX encapsulated in the HALNP sample (“HALNPDOX”). By knowing the amount of DOX as well as the concentration of lipid in the sample, a spectrum of HALNPDOX-PEMs with concentrations ranging from 2.5μg/ml to 0μg/ml DOX were fabricated by varying the concentration of the loaded HALNPs (and thus the total DOX). Secondly, the HALNPDOX particles were adsorbed onto the (PLL/SPS)_4.5_ base platform ensuring the amount of lipid required to achieve a desired DOX concentration was below the saturation conditions determined earlier (5 µg/cm^2). The final mass ratio between Lipid and DOX was 3.125:1. Therefore, for the 2.5 µg/ml DOX concentration samples, 3.9 µg HALNPDOX was adsorbed per well to achieve the desired DOX concentration (well volume = 0.5ml). This similar reasoning was implemented for all lower concentrations of HALNPDOX loading. The only difference in the loading procedure compared to empty HALNPs was the increase in the deposition incubation time to overnight at 4°C to ensure complete embedment as well as protect the activity of the DOX. Supernatant samples were removed following the adsorption protocol to ensure all particles deposited onto the (PLL/SPS)_4.5_ base platform. In addition, DLS and Zeta potential analysis of the HALNPDOX particles was analyzed to validate the encapsulation of DOX did not significantly alter with the size or charge of the HALNPs (and therefore the adsorption process). The (PLL/SPS)_2.5_ capping layer was added immediately after the HALNPDOX adsorption.

21MT-1 cells were seeded at a density of 32,000 cells/well in three 48 well plates with different DOX configurations: 1) PEM platform with embedded HALNPDOX [(PLL/SPS)_4.5_(HALNPDOX)(PLL/SPS)_2.5_], 2) Bolus form HALNPDOX, and 3) free non-encapsulated DOX. After a 24 hours incubation time between the three systems and the 21MT-1 cells, the media was aspirated and 5 mg/ml MTT working solution was added and incubated for 2 hours at 37°C. Cells were then lysed with lysis buffer (acidified IPA) and the absorbance was measured at 570 and 620 nm using a Beckman Coulter AD340 plate reader (Indianapolis, IN, USA). Percent viability was determined by normalization of the 570/620 absorbance ratio to the control untreated cells and positive control dead cells.

**Statistical Analysis.** The difference between experimental groups was analyzed by a one-way analysis of variance (ANOVA) and by a subsequent Turkey’s multiple comparison test in Sigma Plot Software. For statistical analysis of all data, p<0.05 was regarded as the lowest acceptable threshold for significance.


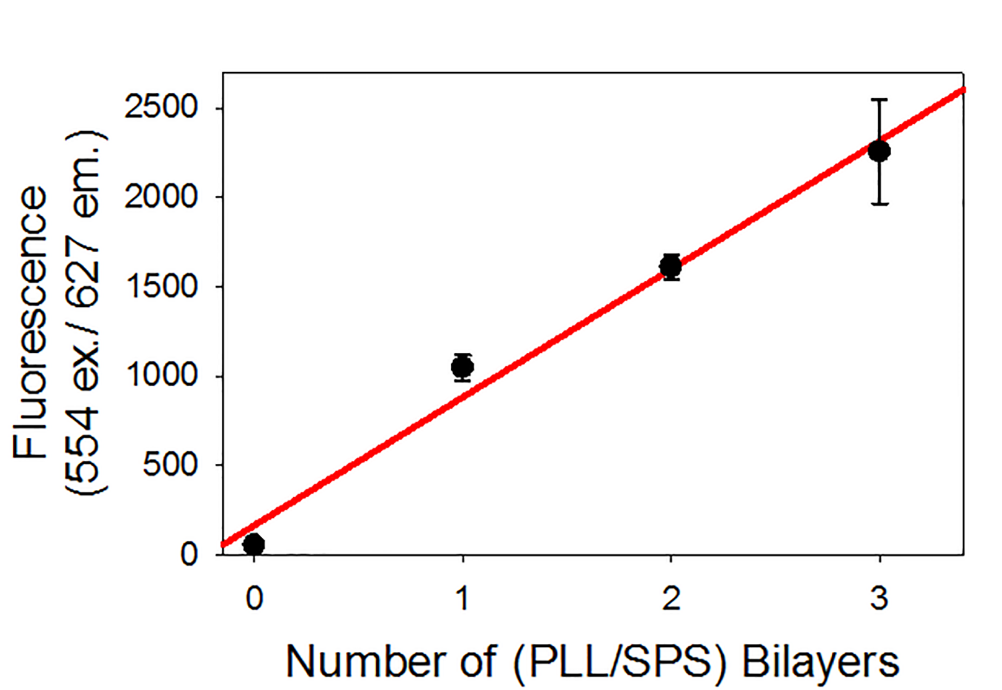


**Sup. Fig. 1.** Fluorescent Monitoring of PEM base formation via Rhodamine tagged SPS


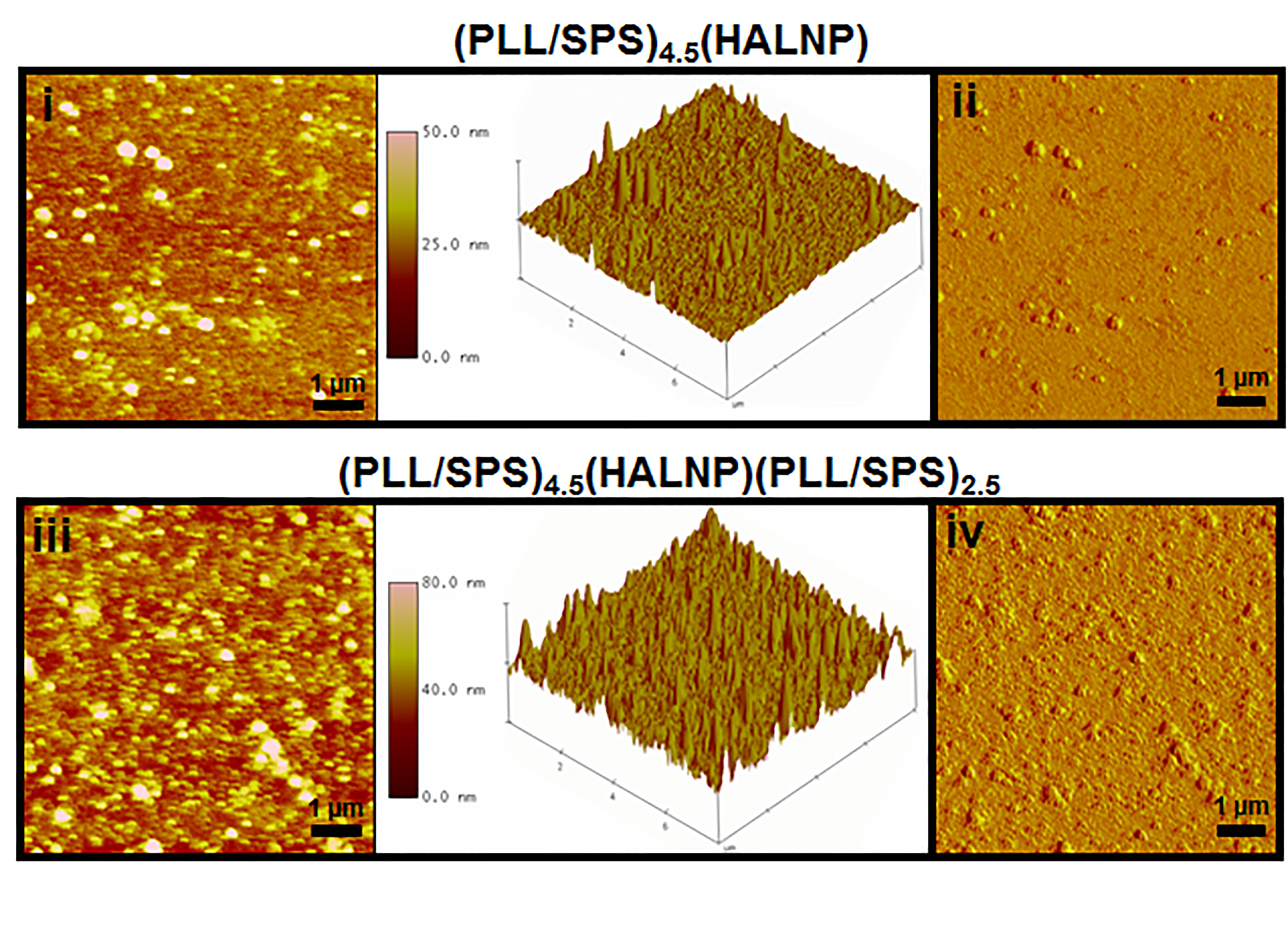


**Sup. Fig. 2.** AFM characterization of HALNPs on the top most layer (i,ii) and HALNPs embedded under the 2.5 bilayers of PLL and SPS “capping layer” (iii, iv). Both height analysis (i,iii) and phase contrast images (ii,iv) were taken for the of the HALNP-PEM systems.


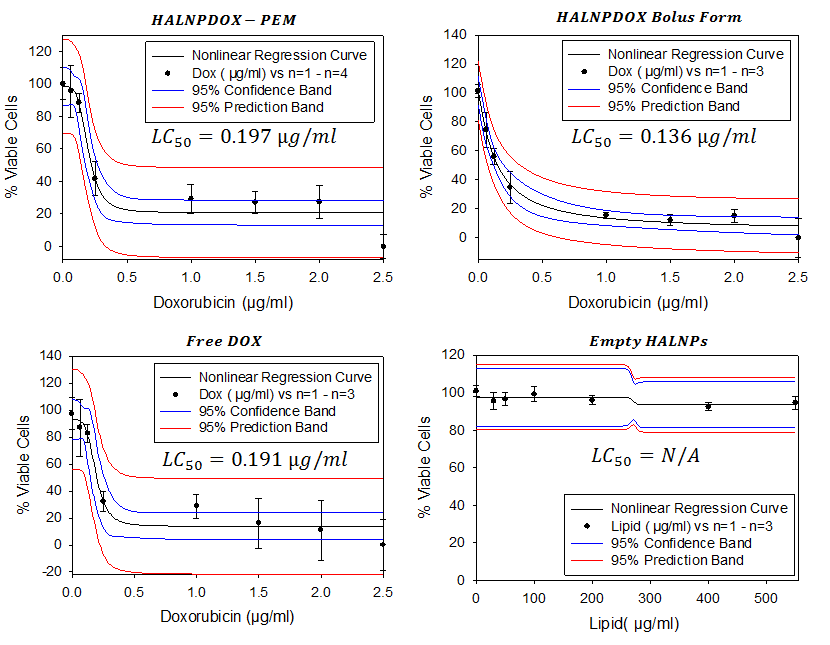


**Sup Fig. 3.** LC_50_ nonlinear regression curves with both confidence and prediction bands for the 24 hr. potency assay comparison between the HALNPDOX Bolus Form, HALNPDOX-PEM, and free form DOX in 21MT-1 metastatic breast cancer cells.


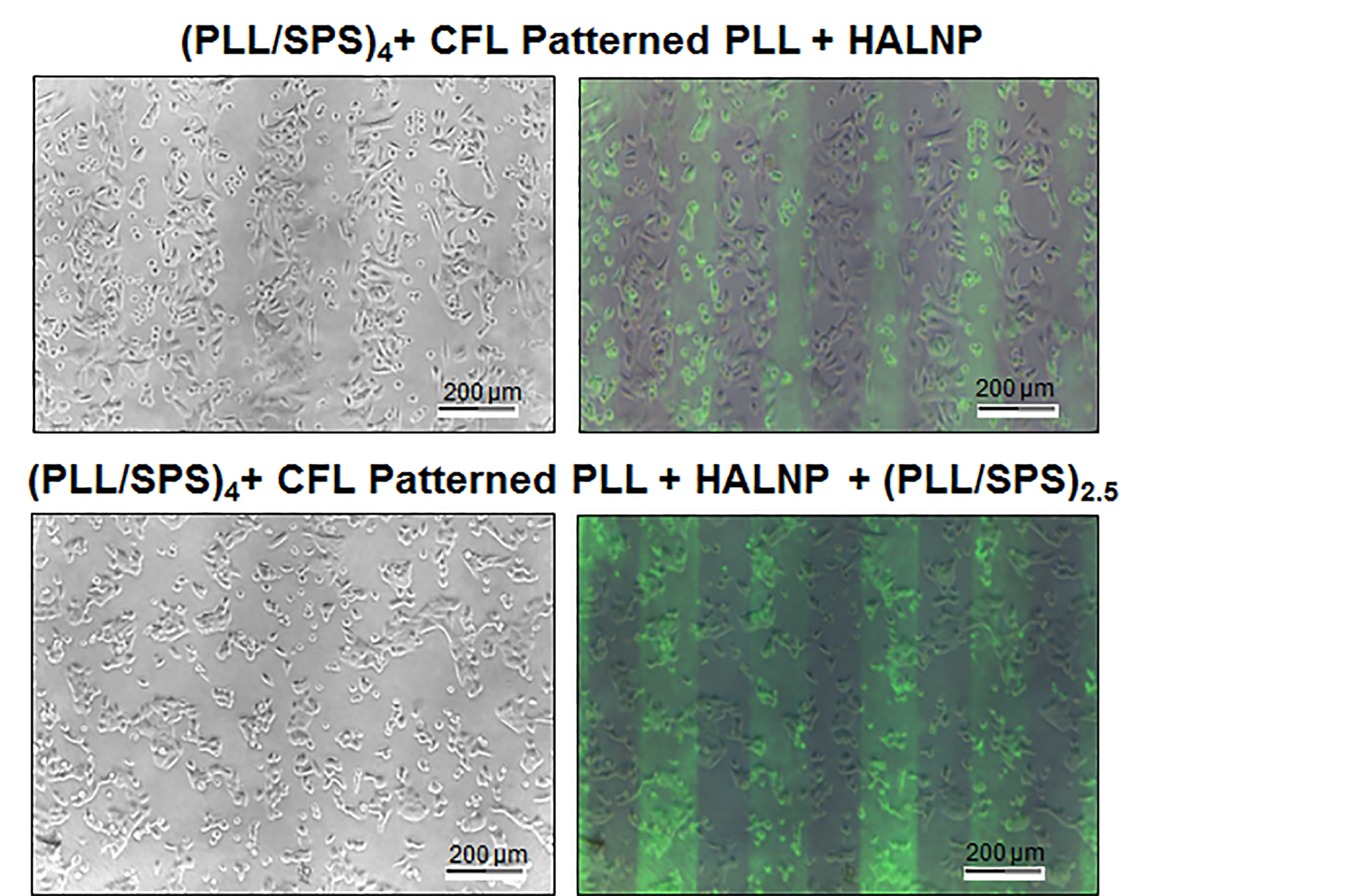


**Sup. Fig. 4** Phase and Fluorescent Microscopy probe of the effect of the capping layer on 21MT-1 cell adhesion 12 hours after cell attachment. In the non-capped sample (A) the cells do not attach to the CFL patterned HALNPs, but instead back fill onto the exposed SPS. In the capped sample (B), the cells cover the HALNP-PEM uniformly due to the homogeneous top most layer of PLL. In either case, the fluorescent uptake only occurs on the cells directly above the patterned HALNPs.

**Graphical Abstract**


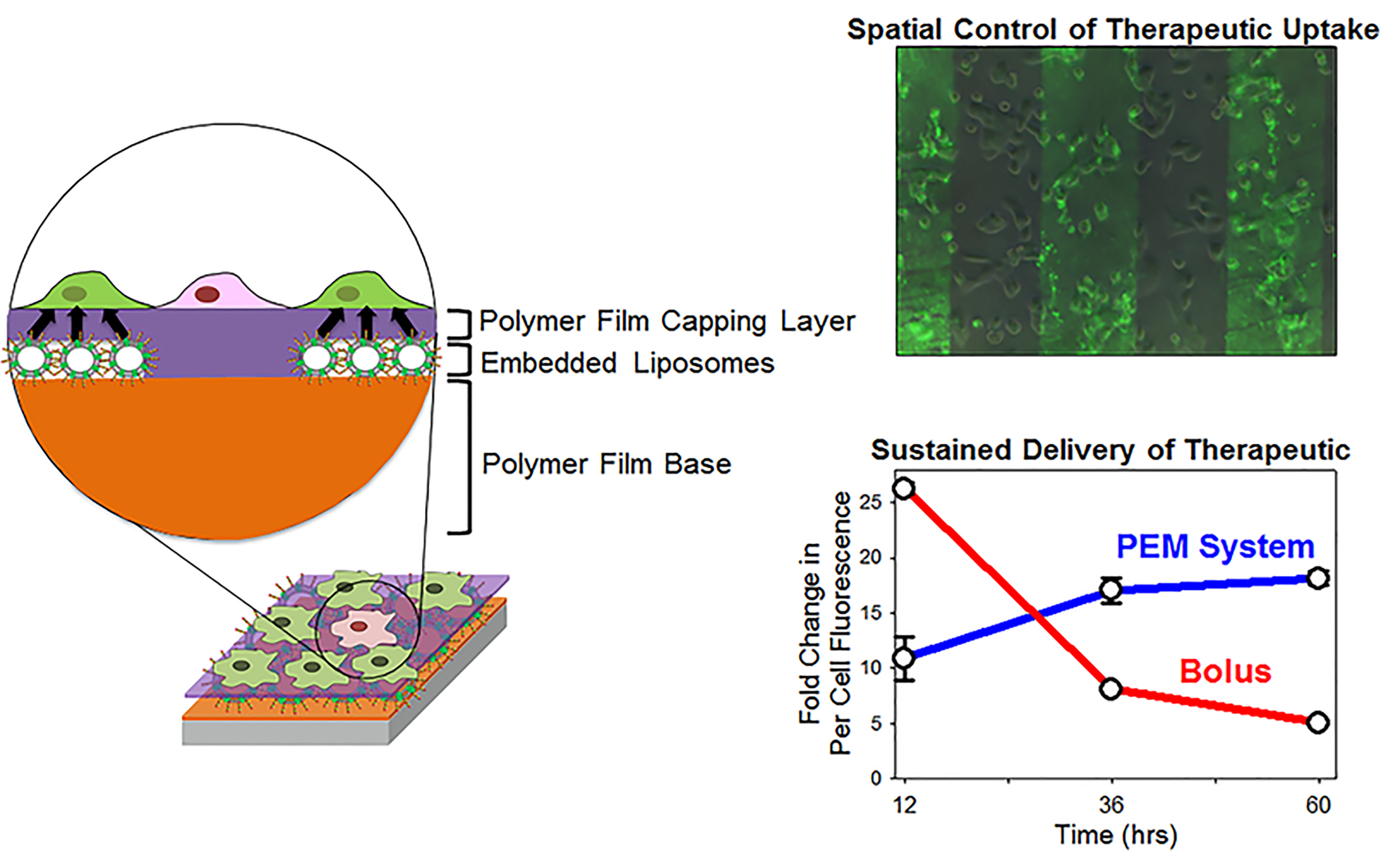


**REFERENCES**

1 Gregoriadis, G. in *Liposomes as drug carriers : recent trends and progress*. (Wiley, 1988).

2 Margalit, R., Okon, M., Yerushalmi, N. & Avidor, E. Bioadhesive liposomes as topical drug delivery systems molecular and cellular studies. *Journal of Controlled Release* **19**, 275-288 (1992).

3 Peer, D. & Margalit, R. Loading mitomycin C inside long circulating hyaluronan targeted nano-liposomes increases its antitumor activity in three mice tumor models. *International journal of cancer. Journal international du cancer* **108**, 780-789, doi:10.1002/ijc.11615 (2004).

4 Peer, D. & Margalit, R. Tumor-targeted hyaluronan nanoliposomes increase the antitumor activity of liposomal Doxorubicin in syngeneic and human xenograft mouse tumor models. *Neoplasia* **6**, 343-353, doi:10.1593/neo.03460 (2004).

5 Peer, D., Park, E. J., Morishita, Y., Carman, C. V. & Shimaoka, M. Systemic leukocyte-directed siRNA delivery revealing cyclin D1 as an anti-inflammatory target. *Science* **319**, 627-630, doi:10.1126/science.1149859 (2008).

6 Srinivasan, C., Peer, D. & Shimaoka, M. Integrin-targeted stabilized nanoparticles for an efficient delivery of siRNAs in vitro and in vivo. *Methods in molecular biology* **820**, 105-116, doi:10.1007/978-1-61779-439-1_7 (2012).

7 Peer, D., Florentin, A. & Margalit, R. Hyaluronan is a key component in cryoprotection and formulation of targeted unilamellar liposomes. *Biochimica et Biophysica Acta (BBA) - Biomembranes* **1612**, 76-82, doi:10.1016/s0005-2736(03)00106-8 (2003).

8 Lynge, M. E. *et al.* Liposomes as drug deposits in multilayered polymer films. *ACS applied materials & interfaces* **5**, 2967-2975 (2013).

9 Martin, C. R. & Aksay, I. A. Microchannel molding: A soft lithography-inspired approach to micrometer-scale patterning. *Journal of materials research* **20**, 1995-2003 (2005).
